# Supplementary figures and images for: mRNA-Seq and miRNA-Seq Analyses Provide Insights into the Mechanism of Pinellia ternata Bulbil Initiation Induced by Phytohormones
Source: Genes (Basel). 2023 Aug 29;14(9):1727. doi: 10.3390/genes14091727 (PMC10531394; doi:10.3390/genes14091727)

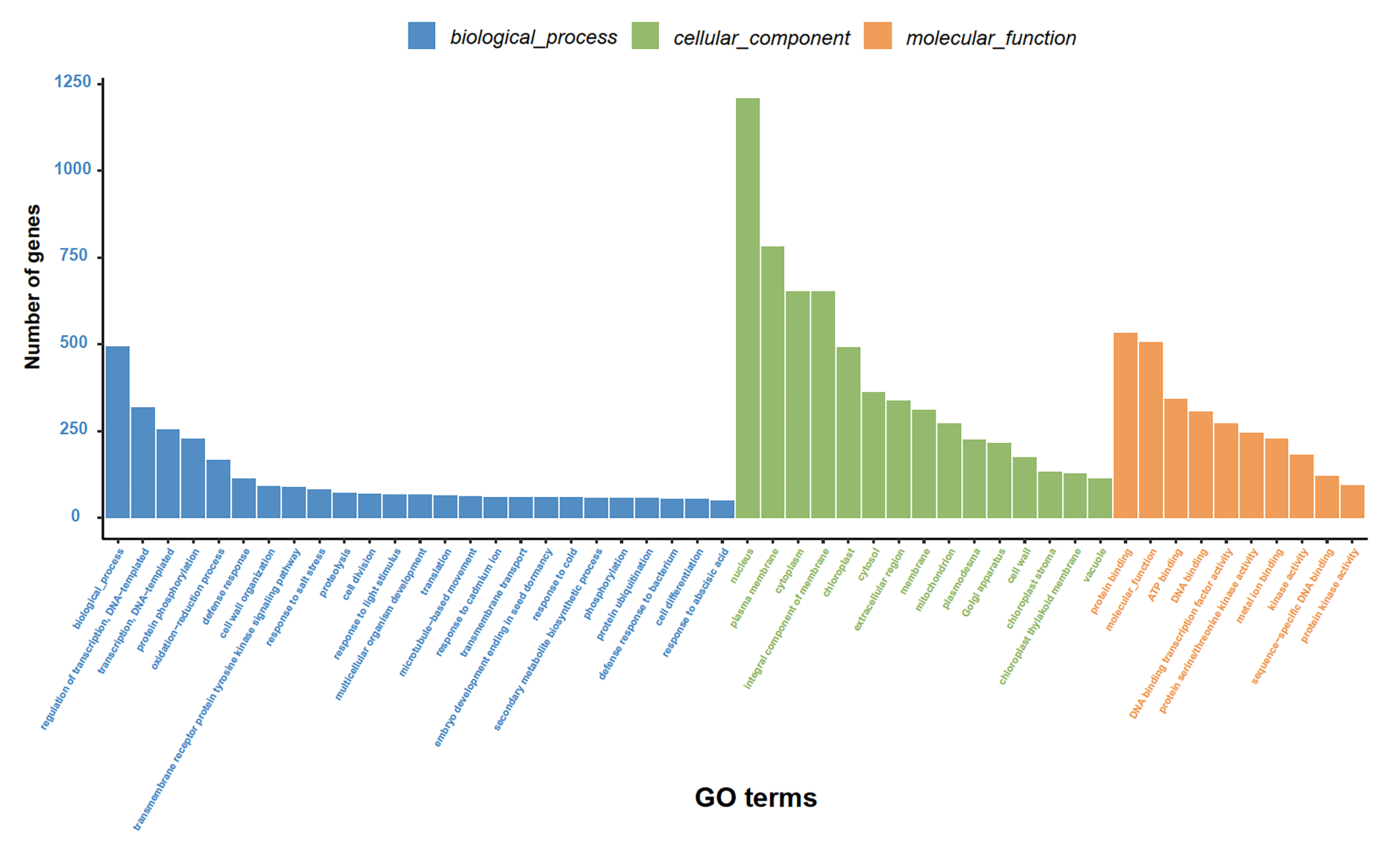

Supplement: Supplementary file 1 [file genes-14-01727-s001.zip › Supplementary File(s)-0823/Figure S1.tif]

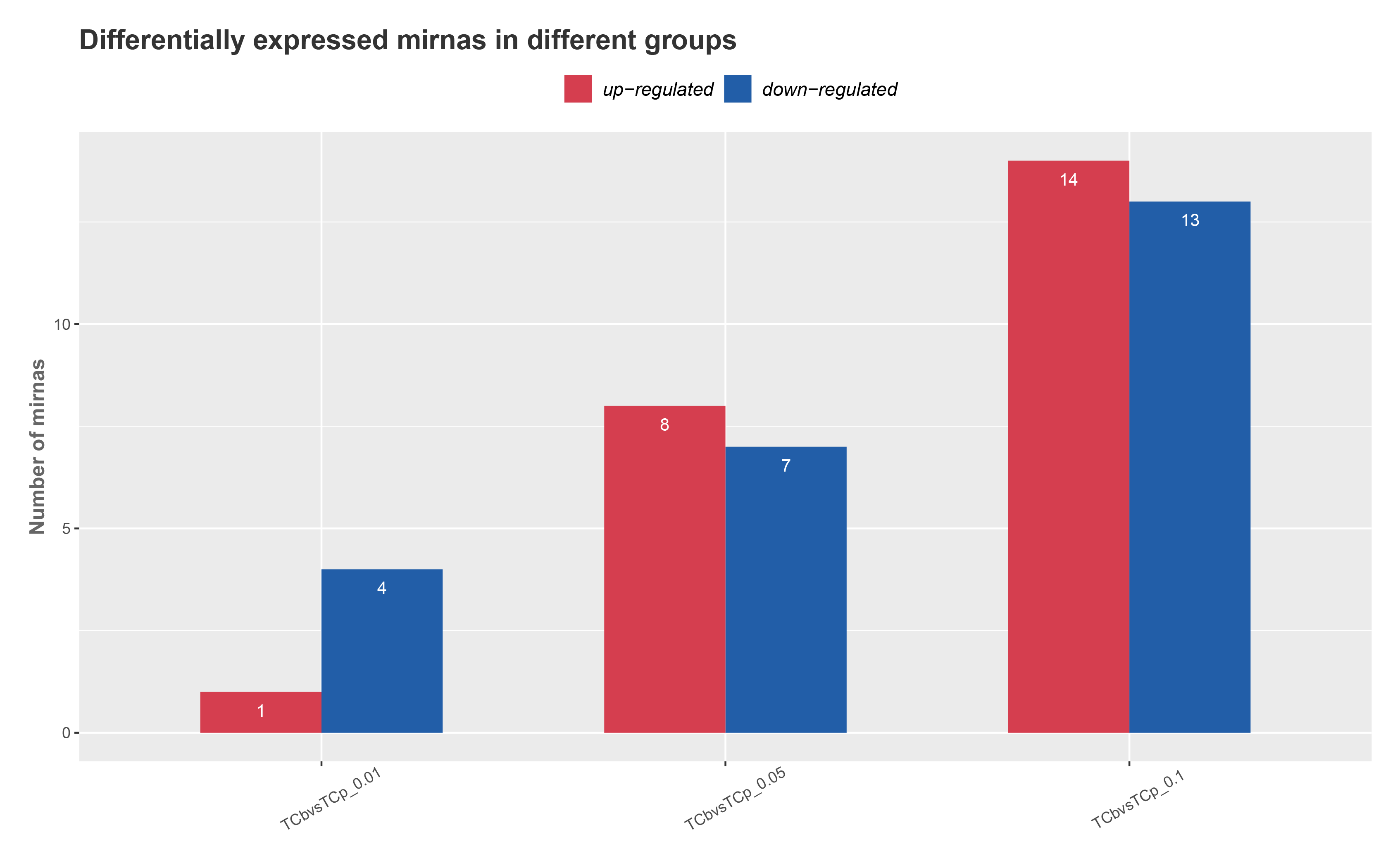

Supplement: Supplementary file 1 [file genes-14-01727-s001.zip › Supplementary File(s)-0823/Figure S2.tif]
